# Improving Access to Behavioral Strategies to Improve Mental Well-being With an Entertaining Breakfast Show App: Feasibility Evaluation Study

**DOI:** 10.2196/25715

**Published:** 2022-03-23

**Authors:** Mariliis Öeren, Iain Jordan, Deborah Coughlin, Sophie Turnbull

**Affiliations:** 1 Method X Studios Ltd Sheffield United Kingdom; 2 Oxford University Hospitals NHS Foundation Trust Oxford United Kingdom; 3 Zinc VC London United Kingdom; 4 Academic Unit of Primary Health Care University of Bristol Bristol United Kingdom

**Keywords:** mental well-being, mental health, smartphone, mobile app, education, entertainment, psychotherapy, feasibility, mobile phone

## Abstract

**Background:**

Although mental ill-health is more prevalent among people from lower socioeconomic groups, digital mental well-being innovations are often developed for people from higher socioeconomic groups, who already have resources to maintain good mental and physical health. To decrease health inequalities and ensure that available solutions are appealing and accessible to people with fewer resources, new approaches should be explored. We developed the app Wakey!, which focused on creating engaging mental health content that is accessible, particularly among lower socioeconomic groups in the United Kingdom.

**Objective:**

The aim of this study is to assess engagement with the app, investigate initial effectiveness data for 6 well-being outcomes, and explore participants’ subjective experiences of using Wakey!

**Methods:**

The app Wakey! was publicly launched on January 20, 2020, and was free to download from Apple Store and Google Play. The app provided its users with entertaining and educational content related to mental well-being. Concurrently, a single-arm mixed methods feasibility trial was carried out from January to April 2020 among people who had downloaded the app and created an account. The primary outcome was engagement, which was collected passively from data logs. Secondary outcome measures were 6 well-being outcomes collected from self-report questionnaires. Individual interviews with 19 app users were carried out in April 2020.

**Results:**

In total, 5413 people fit the inclusion criteria and were included in the final sample—65.62% (3520/5364) women, 61.07% (3286/5381) aged between 25 and 44 years, 61.61% (2902/4710) in employment, 8.92% (420/4710) belonging to the lower socioeconomic group, and 8.09% (438/5413) were engaged users. There was no evidence of a difference in engagement regarding sociodemographic and socioeconomic characteristics. There was evidence that users with a higher average daily sleep score, who joined the study more recently, who had higher baseline self-report of sleep quality, and who found episodes more entertaining were more likely to be engaged users. Among 230 users who provided follow-up data, there was evidence of improvements on four of the six well-being outcomes: life satisfaction (*P*<.001), feeling that life is worthwhile (*P*=.01), ease of getting up in the morning (*P*<.001), and self-efficacy (*P*=.04). The app and its content were well received by those who were interviewed, and several people perceived a positive change in their mental well-being.

**Conclusions:**

This study shows that the app Wakey! could potentially be engaging across different socioeconomic groups, and there is an indication that it could positively impact the mental well-being of those engaged with the app. However, this study was a pragmatic trial with a limited sample, and the selection bias was present in the qualitative and quantitative study. Further work is needed to make any generalizable conclusions.

**Trial Registration:**

ClinicalTrials.gov NCT04287296; https://clinicaltrials.gov/ct2/show/NCT04287296

## Introduction

### Background

Mental health conditions are a considerable burden for patients and health services and have been shown to have social patterning in severity and incidence [1,2]. People in the lowest socioeconomic groups have mental ill-health at higher rates than those in the highest socioeconomic groups [3,4]. Those in lower socioeconomic groups are more likely to be unemployed, working in jobs with low pay, and have insecure work, which have been found to be detrimental to mental health [3,5-7]. Mental health services are struggling to cope with demands on services, and unequal access to support is further exacerbating health inequalities [8,9]. Prevention and broader determinants of health have a larger effect on mental health than reactive, illness-based treatment [10,11]. In addition, people with higher education and higher socioeconomic background who have good access to resources (eg, time, income, and knowledge) are more likely to use commercial mental health solutions in the market to invest in their self-care [12-14].

Digital interventions have been proposed as a solution to address the high demand for mental health support in the context of the crisis in health care services [15]. During the past decade, there has been an explosion of available apps offering mental health and well-being support [16]. These apps target a variety of needs from habit formation to supporting recovery from mental ill-health [17]. Despite the large number of available apps, most lack evidence of effectiveness (ie, no available data) [18] and long-term engagement [19]. Results from a systematic search by Baumel et al [20] show how most apps see a drop in retention between days 1 and 30, 69% and 3%, respectively, depending slightly on the focus of the app (eg, happiness and meditation). Success in engagement has been shown to be a combination of several factors, such as higher rating in app stores, lower price, more positive reviews, good usability, variety in content and features, personalized experience, credibility, high security, social support, and the use of behavior change techniques (BCTs) [21-24]. McKay et al [17] found that 2 BCTs seemed to be more common to use in mental health apps, allowing or encouraging practice or rehearsal in addition to daily activities and providing instructions on how to perform the behavior.

The role of digital mental health interventions in addressing health inequalities is yet to be determined. On the one hand, they provide the potential to reduce health disparities, by providing personalized, low-cost, infinitely reusable resources that can increase access to health interventions [25,26]. On the other hand, they may increase inequity where there remain barriers to access and usability for disadvantaged groups [27,28]. To ensure that health inequalities are not further exacerbated by digital interventions, there is a need to develop and assess digital interventions that manage to maintain long-term engagement and that are appealing and accessible to people from lower socioeconomic groups who often use entertainment to regulate difficult emotions and for education [29-31].

We developed Wakey!, a mental well-being app that is generally appealing across social groups. However, we also aim to address inequalities in access to mental health and well-being support for lower socioeconomic groups in the United Kingdom, by providing content that is both entertaining and led by theory and evidence. The content was developed using information and techniques from cognitive and third-wave psychotherapies, positive psychology interventions, and mental health interventions [32-37]. The web-based psychoeducational strategies are effective at improving mental health literacy [33], reducing stigma [38], and improving the clinical course in depression [39]. Psychoeducational interventions in general are effective (with a small effect size) in managing stress [40].

### Objectives

The purpose of this feasibility study is to explore engagement, assess initial impact, and explore users’ subjective experiences with the app, to inform the next steps. Although we measured the app’s impact on health outcomes, the study was not intended to be a definitive effectiveness trial.

## Methods

### Overview of Study Design

A 12-week mixed methods single-arm feasibility trial was conducted to explore engagement with the app Wakey!, initial effectiveness data, and subjective user experiences. Participants were recruited for the quantitative component exploring the engagement and effectiveness of Wakey! between January 17 and March 30, 2020. The qualitative study to explore people’s experiences of using Wakey! was conducted between April 9 and 24, 2020.

### Ethical Considerations

The trial was approved by the Faculty of Health Sciences Research Ethics Committee at the University of Bristol (reference 98382) and registered in ClinicalTrials.gov (NCT04287296).

### Intervention—The App Wakey!

Wakey! is an app that delivers a 9-minute morning edutainment show, designed as an alternative to an alarm clock (ie, where users can set an alarm and wake up to a breakfast show on their phone). Edutainment refers to media where entertainment is combined with education [41]. The initial pilot of the show (10 episodes) was streamed via Facebook from April to May 2019. During this time, both quantitative and qualitative data (such as desired improvements) were collected and subsequently used as input in the development of the show and the app. Approximately 40.37% (44/109) of the sample had an annual household income below £30,000 (US $40,836.40), which suggests that the feedback received reflected the thoughts and experiences of our target group. In addition, in February 2020, we carried out a think-aloud study among people who lived in a deprived area of London (Southwark and Lambeth). This provided us new insights that influenced usability tweaks in the app, which were implemented before the trial began.

This study focuses on the first 12 weeks after the launch on January 20, 2020, on Apple Store and Google Play. During this time, the show was presented by a comedy drag queen (Ginger Johnson) and a previous cast member from Love Island (Christopher Taylor), who were occasionally accompanied by special guests. Although including a drag queen as a host may turn off some lower socioeconomic group users who may hold more conservative social values, there is evidence that drag queens are seen as an important part in the working-class entertainment in the United Kingdom [42], and people from lower socioeconomic groups tend to watch Independent Television programs, such as Love Island, which is aimed at a less conservative audience [43].

Each week focused on a different mental health–related topic that was discussed by using colloquial language paired with humor in the morning shows (Multimedia Appendix 1). The content was developed (and written by one of the authors (IJ) who is a consultant psychiatrist and psychotherapist) as psychoeducational content designed to communicate the underlying principles from a variety of evidence-based treatments of, and preventative strategies for, common mental disorders. The content was composed of easy-to-understand principles from various cognitive therapies [44], third-wave psychotherapies such as compassion-focused and [45] mindfulness-based therapies [46], positive psychology interventions (such as well-being therapy developed by Fava [34]), and behavioral and lifestyle mental health interventions such as sleep behavior, exercise, and behavioral activation [47]. The content was adapted for the scripts for the show in collaboration with one of the hosts of the show who is a professional writer. Additional content and features were provided on the app to support the theory- and evidence-based techniques, to increase engagement with the app, and to respond to current events (such as the COVID-19 crisis; Multimedia Appendix 1).

### Recruitment

The app was advertised in the United Kingdom through partner companies of the *Lost In TV Audience Services*, who have a database of 450,000 people (approximately 10% were targeted). In addition, the app’s content was advertised through social media platforms (eg, Facebook and Instagram) and more traditional media outlets (eg, *Marie Claire*, *Sunday Mirror*, *The Sun*, and *Metro*).

As this was a feasibility study and focused primarily on uptake and engagement with an interest in the demographics of the users, a sample size calculation was not performed.

### Quantitative Study

#### Procedures

On downloading the app, users were taken through a registration process. Users were presented with the Terms and Conditions and the Privacy Policy, which included consent to enter the study and the data to be used for research purposes (Multimedia Appendix 1). Thereafter, users were asked to provide sociodemographic and socioeconomic information: name, email address, age range, gender, and occupation (Multimedia Appendix 1). Users were asked to set their in-app alarm clock time. Baseline data were then collected on the 4 UK Office for National Statistics (ONS) well-being questions [48], one question inquiring about ease of waking up in the morning and one question inquiring about self-efficacy (Multimedia Appendix 1). Following sign-up, users were taken to the home page (Multimedia Appendix 1), where they were presented with the welcome video.

#### Data Collection

All quantitative data were collected from users via the app. The data on overall engagement were collected passively from data logs on a daily basis (ie, whether users watched that day’s episode or not). Other measures were collected by asking users to provide information at baseline (ie, onboarding) and thereafter on either a daily or weekly basis. Sociodemographic and socioeconomic information was collected only at baseline. A total of 6 well-being outcomes were collected at baseline and then weekly until the end of the trial. Engagement outcomes included the number of people who downloaded the app and created an account, number of shows watched over the 12-week period, average time watched, and entertainment value of the episode (self-reported on a scale of 0 [*not at all*] to 10 [*completely*]).

#### Data Processing

Users were excluded from all analyses if they had not finished creating the account or were aged <18 years. Users whose baseline and follow-up scores were left on the default setting were excluded from the impact assessment. This was 0 for the ONS and sleep questions and *strongly disagree* for the self-efficacy question. In addition, when participants answered *Prefer not to say* to gender (n=49), age (n=32), or occupation (n=703) questions, their answer for a specific variable was treated as missing. Owing to a technical error, 63 users were not able to answer the self-efficacy questions when creating the user, thus missing the baseline assessment and excluded from the analysis about self-efficacy.

Users were segmented into five levels of engagement: never active—had not seen any of the episodes and the welcome video; inactive—had seen only 1 episode or the welcome video or both; became inactive after their first week—saw at least two episodes on their onboarding week and then stopped watching; irregular—had seen at least two episodes on separate weeks but <20% of all the available episodes for them; and engaged users—had seen ≥20% episodes of those available to them. The 20% threshold was a rough equivalent of weekly use—if the user would watch 1 episode per week, then it would equal to 20% of weekly episodes.

The coding of occupational groups is based on two classifications: the Standard Occupational Classification 2010 volume 1 [49] and 2016 ONS National Statistics Socio-economic Classification (NS-SEC) [50]. The 8 NS-SEC categories provided in Table S1 in Multimedia Appendix 1 were used to provide a more detailed overview of socioeconomic groups within the sample of people who used Wakey! during the 12-week trial in the descriptive analysis and to explore the predictors of engagement with Wakey! and improvements on the well-being outcomes.

#### Data Analysis

To see if there were any differences in the probability distributions between the active and never active groups and between people who provided follow-up data and those who did not by sociodemographic and socioeconomic characteristics and content use characteristics (ie, number of watched episodes and entertainment value), chi-square tests and independent-group 2-tailed *t* tests (or Wilcoxon rank-sum tests) were conducted. Wilcoxon signed-rank tests were used to investigate improvements in the 6 ordinal well-being outcomes from baseline to follow-up. To explore predictors of outcomes, binary variables were created, and multiple logistic regression was undertaken, as the parameters were not met for linear regression. For engagement, we explored which variables predicted whether the participant was an engaged user (watched ≥20% of episodes available) or a not engaged user (watched <20% of episodes available). For retention, both the daily (ie, days 1, 7, 14, and 30) and weekly (weeks 1-5) retention were assessed by calculating the proportion of users who created an account and then were active at a specific time. For well-being outcomes, we explored predictors of improvements (≥1 point change) versus no improvement (no change or deterioration). Univariable analysis was conducted using logistic regression to explore predictors of whether the participant was an active user or improved on well-being outcomes. All predictor variables are presented in Table S1 (Multimedia Appendix 2). Repeated-measures multivariable analysis was performed using logistic regression. The initial model inclusion criterion was *P*<.05, with putative predictors entered using backward stepwise selection and retained where *P*<.05. To explore the influence of exposure to Wakey! and to account for different entry times into the study for different participants, we explored associations between improvements in well-being outcomes and user segmentation, the week when they joined the study, if they were an active user, the time between baseline and the last follow-up, and the last week they provided follow-up data.

### Qualitative Study

#### Procedures

The aim was to interview people from lower socioeconomic groups [49,51] and from a diverse range with respect to gender, age range, and user engagement with Wakey! We used purposive sampling [52] and divided people into different groups based on their engagement (to ensure that people from all 4 groups would be represented in the study) and then sent invitations by email (at first) and push notifications to participate in the study (Multimedia Appendix 1). People who were interested in participating in the study received an email with the participant information sheet and a link to the web-based consent form. The web-based consent forms were hosted on the University of Bristol BOS system, and the data were kept in accordance with the Data Protection Act 2018 [53].

#### Data Collection, Processing, and Analysis

Qualitative data were collected via semistructured audio-recorded individual interviews conducted by MÖ. All interviews occurred either on the phone or on a video-call platform. Participants were given a £20 (US $27.22) high-street voucher if they were an active user and £5 (US $6.81) if they were an inactive user as a thank-you for their time. The interview topic guide is outlined in Multimedia Appendix 1. The audio recordings were made on encrypted audio-recorders and transferred to Method X Studios secure servers, where they were kept in accordance with the Data Protection Act 2018 [53]. The anonymized transcriptions were kept separately from the identifiable information on the consent forms, so they could not be linked. Transcribed recordings were anonymized (all names or other identifying material removed), and the collected data were analyzed in themes, which were based on the interview topic guide (using a deductive approach). Gender, age range, and user group have been added to all quotes presented in the *Results* section.

## Results

### Quantitative Study Sample

Between January 17 and March 30, 2020, a total of 5928 people downloaded Wakey! (unique downloads, excluding the Wakey! Team). Of these 5928 people, 515 (8.69%) did not meet the inclusion criteria and were excluded from the study (276 were aged <18 years and 239 had not verified their email address and thus did not finish the registration), leaving a final sample of 5413 users, who were divided into two groups—never active and active. The characteristics of active and never active users are presented in Table 1. Two-thirds of the active users were women (3520/5364, 65.62%) and aged between 25 and 44 years (3286/5381, 61.07%). Approximately 61.61% (2902/4710) of the users were in employment. Approximately 8.92% (420/4710) of the users had an occupation that indicated belonging to a lower socioeconomic group (as defined by the NS-SEC [50]), such as semiroutine and routine occupations (Table S2 in Multimedia Appendix 1). A higher proportion of the never active group was women, in the youngest age group, and not working (unemployed, caregivers, retired, students, looking after family or home, or sickness or disability). In terms of socioeconomic groups, those who were never active were also more likely to have an occupation that indicated them belonging to lower socioeconomic groups.

**Table 1:** Sociodemographic and socioeconomic characteristics among active and never active users.

			Active, n (%)		Never active, n (%)		*P* value	
**Gender (active N=3958, never active N=1406)**								<.001
	Female	2489 (62.89)		1031 (73.33)				
	Male	1404 (35.47)		358 (25.46)				
	Nonbinary or other	65 (1.64)		17 (1.21)				
**Age (years; active N=3972, never active N=1409)**								<.001
	18-24	643 (16.19)		300 (21.29)				
	25-34	1268 (31.92)		459 (32.58)				
	35-44	1195 (30.09)		364 (25.83)				
	45-54	689 (17.35)		236 (16.75)				
	55-64	153 (3.85)		42 (2.98)				
	≥65	24 (0.60)		8 (0.57)				
**Occupation (active N=3455, never active N=1255)**								<.001
	Employed	2191 (63.42)		711 (56.65)				
	Unemployed	137 (3.97)		56 (4.46)				
	Caregivers	241 (6.98)		94 (7.49)				
	Retired	53 (1.53)		10 (0.80)				
	Students	363 (10.51)		184 (14.66)				
	Looking after family or home	301 (8.71)		132 (10.52)				
	Sickness or disability	169 (4.89)		68 (5.42)				
**Socioeconomic groups (active N=3455, never active N=1255)**								<.001
	High	1462 (42.32)		438 (34.90)				
	Middle	414 (11.98)		136 (10.84)				
	Low	290 (8.39)		130 (10.36)				
	Not classified elsewhere	1289 (37.31)		551 (43.90)				

### User Retention and Engagement

Among all 5413 users (this includes both active and nonactive users), 1593 (29.43%) were active on day 1, a total of 273 (5.04%) on day 7, 169 (3.12%) on day 14, 126 (2.33%) on day 21, and 108 (2%) on day 30.

As Wakey! was meant to be used during weekdays and people were onboarded during weekends, it was decided to assess the weekly retention as well. Of the 5413 people, 3135 (57.93%) were active on the week they were onboarded, 1454 (26.87) 1 week later, 635 (11.73%) 2 weeks later, 448 (8.28%) 3 weeks later, and 341 (6.30%) were active 4 weeks later.

The welcome video (length 1 min 26 seconds) was seen by 24.46% (1324/5413) of the users, with a mean watch time of 1 min 6 seconds. The mean number of users watching live streamed episodes was 127 (SD 83.67), and that for archives episodes was 102 (SD 92.22). When combining the unique views of livestream and archived episodes, the mean number of viewers was 219 (SD 131.65). The mean entertainment value of all 60 episodes was 6.80 out of 10 (SD 1.14). Compared with the first week (mean 5.64, SD 0.68), the average score increased by 2.34 points by the last week (mean 7.98, SD 0.43).

When dividing the 5413 users based on the level of engagement, 1420 (26.23%) were never active, 2024 (37.39%) were inactive users, 406 (7.50%) became inactive after their first week, 1125 (20.78%) were irregular users, and 438 (8.09%) were engaged users. The engaged users were divided into those who saw 20%-39% of the episodes available to them (269/5413, 4.97%), 40%-59% (83/5413, 1.53%), and 60% or more of the episodes available to them (86/5413, 1.59%).

In the univariable analysis (excluding the never active users), being engaged users was predicted by a higher average entertainment score (odds ratio [OR] 1.16, 95% CI 1.12-1.20; *P*<.001), a higher average daily sleep score (OR 1.15, 95% CI 1.11-1.20; *P*<.001), joining the study more recently (OR 0.92, 95% CI 0.88-.96; *P*<.001), and a higher baseline report of sleep quality (OR 1.06, 95% CI 1.03-1.10; *P*=.001) (Table S2 in Multimedia Appendix 2). There was no evidence of a difference in engaged users (watched ≥20% of available episodes) versus not engaged users (watched <20% of available episodes) in terms of social characteristics (Table S3 in Multimedia Appendix 2). The results from the multivariate model are presented in Table S4 (Multimedia Appendix 2).

### Effectiveness on Well-being Outcomes

When comparing users (N=3993) who had seen at least one episode (or the welcome video) and provided follow-up data with those who had seen at least one episode (or the welcome video) and had not provided any follow-up data, there was a higher proportion of users aged ≥45 years and users who were from middle socioeconomic groups and who provided follow-up data on ONS, sleep, and self-efficacy measures (Table S5 and Table S6 in Multimedia Appendix 2). In addition, users who provided follow-up data had rated episodes with a higher score (mean=7.5 vs 5.2; *t*_1348_=−8.6134, *P*<.001) and had watched more live (11 vs 1; *t*_3885_=−36.1515, *P*<.001) and archived episodes (6 vs 1; *t*_3885_=−26.9019, *P*<.001).

Users who provided follow-up data demonstrated improvements on 4 of the 6 health outcomes (Table 2). There was strong evidence (*P*<.001) that users experienced improvements on life satisfaction and ease of getting out of bed in the morning (median difference 2 points). There were also improvements in feeling that life is worthwhile (median difference 1, *P*=.01) and in self-efficacy (median difference 0, *P*=.04).

**Table 2:** Evidence of improvements in outcomes from baseline to final follow-up (N=5413).

	Users whose score improved		Users with baseline data, n (%)		Median at baseline (range, IQR)^a^		Users with follow-up data, n (%)		Median at follow-up (range, IQR)^a^		Difference from baseline to follow-up		*P* value
	N	n (%)											
Life satisfaction	230	112 (48.7)	3638 (67.21)	6 (0-10, 4-7)		230 (4.25)		7 (0-10, 5-8)		+1		<.001	
Worthwhile	230	111 (48.3)	3638 (67.21)	6 (0-10, 4-8)		230 (4.25)		7 (0-10, 5-8)		+1		.01	
Happy yesterday	230	97 (42.2)	3638 (67.21)	5 (0-10, 4-8)		230 (4.25)		6 (0-10, 4-8)		+1		.43	
Anxious yesterday	230	97 (42.2)	3638 (67.21)	5 (0-10, 2-7)		230 (4.25)		4 (0-10, 2-7)		−1		.73	
Easy to get up	230	133 (57.8)	3638 (67.21)	4 (0-10, 2-7)		230 (4.25)		6 (0-10, 4-8)		+2		<.001	
Self-efficacy	158	53 (33.5)	3577 (66.08)	4 (1-5, 3-4)		158 (2.92)		4 (1-5, 3-4)		0		.04	

^a^Baseline and final follow-up data were collected at different time points for different users, as people were able to sign up at any point during the 12-week trial, and follow-up data were collected each week. Follow-up data were reported as the final outcome data reported by the users.

There was a single variable that predicted improvements in life satisfaction (*P*<.05) in the univariable analysis; therefore, no multivariable analysis was conducted (Table S7 in Multimedia Appendix 2). Lower self-efficacy at baseline was associated with improvement in life satisfaction by the end of the trial (OR 0.63, 95% CI 0.50-0.80; *P*<.001).

There were 2 variables associated with improvements in feeling life is worthwhile by the end of the trial. Those participants who improved on perceiving the life worthwhile were more likely to have a lower baseline sleep quality (OR 0.89, 95% CI 0.81-0.97; *P*=.01) and have had lower self-efficacy at baseline (OR 0.78, 95% CI 0.63-0.98; *P*=.03). The results from the multivariate model are presented in Table S8 (Multimedia Appendix 2).

Four variables were associated with improvements in sleep quality in the univariable analysis: higher number of archived episodes watched (OR 1.04, 95% CI 1.00-1.08; *P*=.04) and lower baseline scores for three of the ONS questions—satisfaction with life (OR 0.83, 95% CI 0.74-0.93; *P*=.002), life being worthwhile (OR 0.77, 95% CI 0.69-0.87; *P*<.001), and happiness (OR 0.85, 95% CI 0.76-0.94; *P*=.003).

A single variable was associated with self-efficacy in the univariable analysis; those with a lower baseline score of perceiving life being worthwhile (OR 0.86, 95% CI 0.76-0.99; *P*=.03) were more likely to show improvements in self-efficacy.

### Qualitative Study Sample

In total, 1524 people received an invitation to participate in the qualitative study, and of these 1524 people, 76 (4.99%) were interested in participating. Ultimately, 1.25% (19/1524) of the users consented, all of whom were interviewed. Of the 19 participants, 9 (47%) were women, 12 (63%) were aged between 25 and 44 years, and 15 (79%) were not in full-time employment (eg, student, caregiver, and unemployed). On the basis of the segmentation of the level engagement, of the 19 participants, 9 (47%) were active users, 5 (26%) were irregular users, 2 (11%) had become inactive, and 3 (16%) were inactive users.

### Qualitative Results

#### Hearing About and Using the App

Participants mainly heard about Wakey! through social media, such as Facebook and Instagram. When asked about what made the interviewees interested in the app, different reasons were mentioned, such as having a drag queen as one of the hosts, having a different approach to waking up, and addressing mental health issues (almost all interviewees reported having mental ill-health currently or in the past).

When asked about how they used the app, most used Wakey! as part of a morning routine after waking up or sometimes later during the day, rather than as an alarm clock. Whereas some people had seen change in their use of Wakey! during lockdown, others had stuck to their routine or way of life.

There’s not much difference for me, to be honest, because I’m isolated anyway...There are a lot of people with physical disabilities and mental disabilities and illnesses that live like this already.

Female, aged 35-44 years, active

The interviewees who were active users said that they watched Wakey! on most mornings or every day (including watching old episodes during the weekend or watching archived episodes if they missed the livestream ones). When asked why they might have missed some of the episodes, the main reasons were the change in their morning routine due to lockdown (eg, change in work times and not having to wake up at a particular time), forgetting to watch the show, and occasionally oversleeping or staying up late. The use of the app varied slightly among the irregular users. Of them, 4 were watching the show at least three times a week, including occasional watch of the archived episodes. Getting late to bed or forgetting about the show were two reasons behind not watching the show more frequently. One of the irregular users felt that sometimes he feels it was too overwhelming to watch the show.

To be honest with you, it’s how I feel on the day...It is basically about trying to be in the right state of mind...trying to be in the mood to watch Wakey Wakey is one thing because sometimes if you're not feeling too great within yourself, it’s like the last thing you want is sometimes to be looking at some people, where everyone's laughing, because it’s hard.

Male, aged 25-34 years, irregular

Watching Wakey! was usually less regular among people who became inactive after their first week or who had been inactive before the qualitative study. Some of them mentioned that they tend to watch Wakey! a couple of times a week or as often as possible. The reasons behind not watching the show more often were related to the change in their routine during lockdown (as among active users) and work routine.

#### The Presenters and Content

Interviewees’ general impression of the app was positive. They were very satisfied with the presenters, and it was mentioned several times that they seem very friendly and have a great chemistry, which makes the show very enjoyable. Several people pointed out that the content of Wakey! was very well developed, as it covered a variety of useful topics on mental health and provided practical tips that people can relate to and incorporate into their lives, and it was done in a fun and entertaining way that helped keep people engaged.

You’ve wrapped it up in an entertainment bubble, but it’s very much about mental health. I think it’s brilliant the way it’s done.

Female, aged 35-44 years, active

I think it’s really good that they’re focusing on a lot to do with mental health because that is a big thing that gets pushed under the carpet. There’s so many of us that look okay on the outside but might be suffering.

Female, aged 35-44 years, irregular

#### Use of Features

Several interviewees had used the chat option during livestream episodes, Q and A sessions, or *Brain–aerobics* quizzes. They liked that using the chat created the feeling of a community through interacting with other users. In addition, it was appreciated that people received responses to their questions and comments.

I do appreciate the fact that somebody from Wakey! has actually replied to me and had a bit of a conversation with me. I do like the interactive aspect and getting involved with people.”

Female, aged 25-34, active

By the time the interviews occurred, 8-9 articles were made available on the app. Whereas some people had read the articles and found them useful, most had not yet engaged with them.

There were two types of live events: Q and A and *Brain-aerobics* quiz. Not everyone was aware of the quiz, but those who were viewed it positively, describing it as an opportunity to interact with Ginger Johnson and other users. Only a small number of interviewees had watched Q and A live events (as people had other responsibilities at the time it was broadcast). Of those who had, some found them useful, whereas others did not feel they were relevant to them or found them boring.

Among users who had visited the progress page, the regularity of use varied; however, in general, people found it useful as a tool to track their progress and see improvements.

#### Data Collection

Daily questions at the end of episodes were perceived, among some participants, as a possibility for self-reflection. Whereas some of them would have liked more daily questions, others found them too frequent. Whereas the majority remembered the daily questions, several struggled to remember if they had answered the weekly ones. Those who had answered the questions commented that the questions can be useful when you are interested in your mental well-being and show how things have changed compared with previous weeks.

When you actually put that number in on the score, you think to yourself, “I actually feel a lot better than what I thought,” or, “I actually feel a lot worse than what I thought.” I think it’s a good idea definitely because it helps well, yourself to be able to see how you are feeling.

Male, aged 25-34 years, irregular

When I joined, I was quite shocked by doing the survey at the end, when you have to give yourself points as to how you feel and things. It shocked me how bad I was feeling. I hadn’t realized that that’s how I was feeling.

Female, aged 55-64 years, active

#### Perceived Change in Outcomes

When asked about changes in mood, sleep, or other similar aspects that might relate to using Wakey!, almost all interviewees had some positive examples of change that related to improvement in their mood, paying more attention to having a routine (which was perceived as likely to have an effect on sleep quality), finding it easier to getting up from the bed in the mornings, and reduced stress levels.

Using the app made me feel better in the morning, got me up, got me alert, got me awake, relatively easy. Whereas I previously tended to snooze my alarm clock frequently.

Male, aged 45-54 years, irregular

It just gets me out of bed at a proper time. I’ve noticed that in personally myself, I’ve been able to smile a bit more rather than being a bit down in the dumps. Even if I am in a bit of a mood when I wake up, by the time I’ve watched an episode of Wakey!, I’ve giggled me up for a good 10 minutes.

Male, aged 35-44 years, active

#### Usability Issues and Future Improvements

Although people had many positives to say about the content of Wakey!, there were things that participants felt could be improved, such as some users feeling that the show felt a bit rushed and too preplanned and some of its content repeating itself. Technical issues mentioned included the following: alarm not going off, videos freezing, issues with chat’s functionality, livestream episodes not starting from the beginning, and not being able to save the videos or watch them offline.

When interviewees were asked about three things they would keep if everything else about Wakey! would be changed, the three most popular things were the presenters and the Old News and *Bed-Aerobics* segments from the morning shows (described in Multimedia Appendix 1).

That’s a tough one. Things to stay, obviously, first of all, is the presenters. Secondly, I’d say the content...is fantastic.

Male, aged 25-34 years, active

Several participants found it difficult to answer but instead focused on things that could be improved. Suggestions included addressing different parts of the app, such as the content, survey, chat, live events and challenges, length and frequency of the episodes, and technical issues. One of the interviewees emphasized that the app’s objective is not entirely clear and could have an effect on people not using it.

I think one of the main things is it’s not entirely clear what the app’s meant to do. It’s a wake-up alarm clock, and the mental health and well-being side of it’s not that clear until you’ve started using it.

Male, aged 45-54 years, irregular

## Discussion

### Principal Findings

This study has three main objectives: (1) exploring engagement, (2) assessing the app’s potential to improve mental well-being, and (3) exploring users’ subjective experiences with the app. The findings provide insights into the potential an edutainment app can have, from both the engagement and effectiveness perspectives. Fleming et al [19] have shown that the use of digital interventions can be more modest when launched in a real-world setting (compared with a trial setting). As Wakey! was made publicly available to everyone, the findings are more likely to reflect the *real-world* data and to be ecologically valid. Although 8.09% (438/5413) of the users were considered as engaged, it is difficult to compare the level of activity at Wakey! with other apps, owing to differences in definitions and the content [19]. For example, moderate use can be viewed as using an app 1 week after installation, but it can also mean that a proportion of users (7%-16%) have completed at least two modules on the app [19]. In contrast, there are universal indicators such as user retention that enable the initial comparison. Baumel et al [20] showed that, on average, around 70% of mental health apps’ users are active on day 1. That number drops significantly and reaches 10% by day 7 and 4% by day 30. When comparing the user retention numbers of Wakey! with the average, the former are around twice as low at each time point. At the same time, Wakey! is a weekday app, with no new content added during the weekend, and as the expectation for an active user was to use the app at least once a week, it was decided to see the weekly retention as well. Although the week 1 retention of Wakey! is still lower than the average day 1 retention, user activity does not drop that rapidly in the following weeks. Although days 1, 7, 14, and 30 retention rates tend to be more common to use [20], the authors would recommend considering using weekly retention as an accompanying metric, especially if the app is not meant for daily use.

Although Wakey! aims to decrease inequalities by increasing access to mental health information and products to people from lower socioeconomic groups, only a small proportion of users were from these groups. In addition, people who had never used the app were more likely to be from lower socioeconomic groups. Taken together, these findings suggest that the version of Wakey! explored in this trial was less accessible or appealing to people from lower socioeconomic groups. Low uptake can be influenced by a variety of factors, such as low digital literacy, lack of awareness of the app, low availability and accessibility, lack of recommendations from other people to use the app, and lack of support to navigate new technology [54,55]. The difference in use has been explored in several systematic reviews, but the evidence regarding socioeconomic characteristics is inconclusive. For example, Turnbull [56] found that people with higher income were more likely to use digital solutions that addressed chronic health conditions, but there were no differences in use regarding education and employment. However, it was highlighted that caution should be taken with conclusions drawn from these findings because the number of studies included in the analysis was small and there was a high risk of bias. Beatty and Binnion [57] investigated the adherence to web-based psychological interventions and found that in 28% of studies reporting education, higher adherence was predicted by higher education. Similar to Turnbull [56], the authors did not find any association between employment and adherence in all studies reporting employment. Perski et al [58] found that higher levels of education and employment were associated with greater engagement with digital behavior change solutions.

On the basis of the responses from a small number of users who provided qualitative data, participants’ general experience with the app was positive. This was in agreement with the high average entertainment scores reported in the quantitative data. The latter, in turn, was one of the indicators that predicted higher engagement with the app. The results of this study also indicate that in the small sample (n=158-230) of users who were engaged with the app and provided data for health outcomes, improvement was seen across different mental well-being outcomes, such as life satisfaction, life being worthwhile, ease of waking up and self-efficacy, and users’ perception of positive change in their mental well-being. A recent systematic review highlighted that only 2% of publicly available psychosocial wellness and stress management mobile apps have published peer-reviewed evidence of feasibility or efficacy [18].

Mental health apps vary by different factors, such as the mental health conditions they target [59,60] and the BCTs used [17]. For example, providing instructions on how to perform a behavior and allowing or encouraging practice or rehearsal in addition to daily activities are the two most popular BCTs used in apps that aim to improve mental well-being [17]. The content of Wakey! has influences from different types of therapies and psychology interventions and covers a variety of BCTs shown to have an impact on mental well-being [17,61]. Although the concept that Wakey! uses—mixing entertainment with education—has been used for decades in various forms (eg, *Sesame Street* in the Unites States and *The Archers* in the United Kingdom) [62], its uniqueness largely stems from its format—it is about addressing mental well-being at the start of the day through a breakfast show. However, the app should be more appealing to the target population who already tends to be less likely to use digital health solutions [63-65].

There are several ways to make the app more appealing to people from all socioeconomic groups. Our qualitative findings in this study suggested that not everyone was aware of the app’s objective when they downloaded it (ie, a focus on improving mental well-being) and providing a clear description of the app in social media and in the App Store and Google Play is “a key channel to inform consumer choice” [66]. For some people, this led to dissatisfaction and disengagement because they were seeking entertainment, not entertainment and support. We will seek to clarify the description of the app in the App Store and Google Play, to improve clarity of the purpose of the app, and to reduce misunderstanding about its purpose. To make the app more appealing and user-friendly to lower socioeconomic groups specifically (as we did not quite reach the groups expected), further work (eg, cocreation workshops and feedback sessions) is being undertaken with the target group. These methods are evidenced to improve the accessibility of behavior change interventions and to improve engagement in these groups [67,68].

Huang and Bashir [21] investigated how information cues across anxiety apps influence the selection and adoption of the app. They found that users were more likely to select apps that were cheaper and had better ratings and reviews. Schueller et al [69] asked people to rate the importance of health apps’ features and found that content, ease of use, and cost were the most important factors regarding uptake and continued use. Alqahtani and Orji [22] found that poor usability, unvaried content, and lack of personalization were the most common reasons why users stop using mental health apps. Although the general experience of the app described by the sample of qualitative study participants (n=19) was positive (eg, easy to navigate, useful, and entertaining content), it was also pointed out that technical issues should be addressed, and the content could be more varied. Although there is a lack of information regarding the main reasons why people stopped using the app, several interviewees mentioned the issue of the alarm not working properly, and similar feedback was also received via emails and morning chat. Thus, our best guess is that functional issues with the alarm could be one of the main reasons why people stopped using the app (ie, becoming inactive). The app was advertised as an alternative to a traditional alarm clock, and the feedback received implied that it was not fully functional for everyone—the phone had to be unmuted and connected to the internet.

Uptake and ongoing use of apps are influenced by a variety of factors, which are often related to users’ needs and resources. To reduce barriers to access for those with lower incomes, Wakey! is designed to be free to use and the entertaining content becomes available as soon as the user creates an account.

### Limitations

First, the study lacked the comparator group. It is therefore not possible to know if improvements in the 4 well-being outcomes were related to the use of the app.

Second, the sample size of people who provided follow-up data is small, and this limits the generalizability of the findings. Although there was an indication that these users improved on 4 health outcomes, these results should be interpreted with caution as the data are likely to be not missing at random [70]. Users who provided health data had rated episodes with a higher score and had watched more episodes; a higher proportion were aged ≥45 years and were more likely to be from a middle socioeconomic group. Therefore, this group of individuals was not representative of the whole sample but can be taken of an indication that those engaged with the app can benefit from its use. Using incentives (eg, quizzes and prizes) would be an option to motivate people who do not normally engage in research to provide data.

Third, the app was targeting people from lower socioeconomic groups, but the group was underrepresented in the final sample (420/4710, 8.92%).

Fourth, more than one-third of users (1840/4710, 39.07%) who provided data on their occupation belonged to a group *Not classified elsewhere*. This group included people who were not in employment—unemployed, caregivers, retired, students, looking after family or home, or sickness or disability. It is possible that a proportion of these people who had engaged with Wakey! would have been classified as people from lower socioeconomic groups, as those people who are not in employment may have lower levels of access to resources. However, because we did not have any information about people’s income or prior occupations, it was not possible to infer their socioeconomic group in this study. In a future study, prior occupations and other indicators of access to resources would be sought to support the identification of the socioeconomic group.

Fifth, the frequency of follow-up reports varied among the participants, from 35.2% (81/230) of the people whose last follow-up was after 1 week of using Wakey! and 5.2% (12/230) from the 12th follow-up week. To account for this, we explored the influence of study entry dates and the dates of follow-up data in the analysis of predictors of improvements in outcomes. As only a small number of variables were captured to reduce the data burden on the app’s users, it is likely that several important covariates that affected participants’ mental well-being were not captured, including social characteristics (such as ethnicity) and mental health indicators.

Sixth, 3 of the 6 well-being questions asked were based on recent past (how happy and anxious you were yesterday and how easy it was to get up this morning) and might not reflect users’ overall state.

Seventh, there was a lower proportion of users who were less engaged with the app (inactive or who had become inactive user groups) in the qualitative study compared with those who were more engaged (ie, active and irregular user groups). Although we used a diverse range of recruitment methods (eg, sent emails and push notifications and used incentives) to include an equal number of participants from each group, we found it very challenging to get inactive users to engage with the qualitative study. Ultimately, we interviewed everyone who expressed interest by the end of the study, thus potentially introducing self-selection bias [71]. We appreciate that this limits the conclusions that can be drawn from this study as we did not reach data saturation and interviewed those who were more motivated to share their experience. Interviewing more inactive people might have provided us some additional insights that potentially may have diverged from our sample.

Eighth, the occurrence of the COVID-19 lockdown may have had an impact on the study outcomes and user engagement with the app.

### Conclusions

Digital mental well-being solutions are often aimed at people from higher socioeconomic groups, and the majority struggle with high drop-off rates. This study shows that an app could not only be potentially engaging across socioeconomic groups if its content is grounded in theory and evidence but also be engaging and entertaining. There is also an indication that this type of app can have a positive impact on mental well-being among more engaged users.

However, this study was a pragmatic trial, which was based on a limited sample without a control group, and these results apply to the particular group of people in the study. Thus, it is not possible to generalize the results to a wider population.

As we saw that the uptake of the app was significantly lower in lower socioeconomic groups, further work that involves cocreation with the target group is to be undertaken.

## Appendix

Multimedia Appendix 1 Supporting information: the app, socioeconomic groups, qualitative study.

Multimedia Appendix 2 Supporting tables (sociodemographic and socioeconomic characteristics, variables included in analysis, multivariate logistic regression models, and univariable associations).

## Abbreviations

BCT: behavior change technique
NS-SEC: National Statistics Socio-economic Classification
ONS: Office for National Statistics
OR: odds ratio
