# Supplementary material for: Improving Access to Behavioral Strategies to Improve Mental Well-being With an Entertaining Breakfast Show App: Feasibility Evaluation Study
Source: JMIR Form Res. 2022 Mar 23;6(3):e25715. doi: 10.2196/25715 (PMC8987957; doi:10.2196/25715)
Supplement: Multimedia Appendix 1 [file formative_v6i3e25715_app1.docx]

**Multimedia Appendix 1 – Supporting information**

[The format and the content of the breakfast show 2](#_Toc92292534)

[Additional content and features of the app 2](#_Toc92292535)

[Terms and Conditions 5](#_Toc92292536)

[Privacy Policy 7](#_Toc92292537)

[Personal data collection 9](#_Toc92292538)

[Socio-economic groups based on occupation 10](#_Toc92292539)

[The App – Home page 12](#_Toc92292540)

[Qualitative study procedures 12](#_Toc92292541)

[Interview topic guide 14](#_Toc92292542)

# The format and the content of the breakfast show

*Format*

Users had access to two types of episodes – livestream and archived shows. The livestreamed episodes were available between 6–9AM from 20 Jan – 28 Feb and between 5–9AM from 2 Mar – 10 Apr. There was a live chat that was available while these episodes were streaming which were moderated by a Wakey! team member. The livestream was available on a loop of 180 (until 28 Feb) and 240 minutes (from 2 Mar), with a varying waiting time between the episodes (the waiting time was 5–6 minutes for the first 10 episodes and thereafter ~30–60 seconds). Depending on the length of the show (and when users started viewing) they would access into the show at a fixed time – for example, if they started viewing at 8AM they’d see the start of the show, if they started viewing at 8:03AM they’d see the show 3 minutes in. A member of the Wakey! team started the loop each day just before the show started. The variance of timing was within 10 seconds, to minimise differences in experience from the users’ perspective. After livestreaming finished around 9AM, the episodes went into archive and users could watch them retrospectively, with no live chat option.

*Content*

Each week focused on a different mental health related topic (decided on by a team of postdoc researchers, creative producers, and a psychiatrist) that were discussed in a fun and entertaining way. The first 12 topics (in order) were: change and growth, energy and exercise, connection, validation / self-compassion, behavioural activation, facing fears, rumination, jobs, confidence and self-esteem, endings and moving on, wellness, and routine and isolation. In addition to the mental health topics two segments were covered in the shows: Bed-Aerobics (where Chris and Ginger did light exercises in bed) and Old News (this segment focused on interesting facts from the past).

# Additional content and features of the app

Additional content and features were provided on the app to support the theory and evidence-based techniques, to increase engagement with the app and to respond to current events (such as the COVID-19 crisis), including:

- in-app live events (e.g. Q&As with the team’s psychiatrist, supporting users through the pandemic, “Brain-Aerobics” quizzes with Ginger Johnson),
- engaging activities (episode introductions by Wakey! users, challenges and competitions),
- a moderated live chat while the episode was streaming,
- weekly in-app articles,


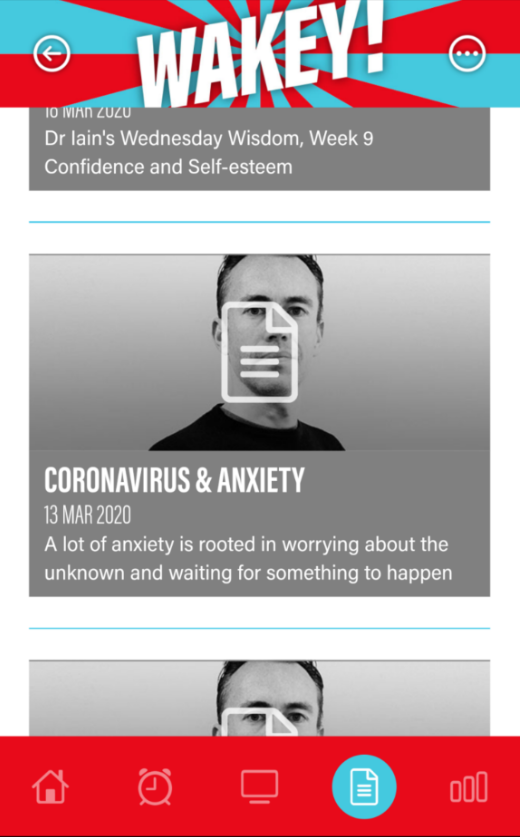


- weekly newsletters via email covering the main topic of the week,


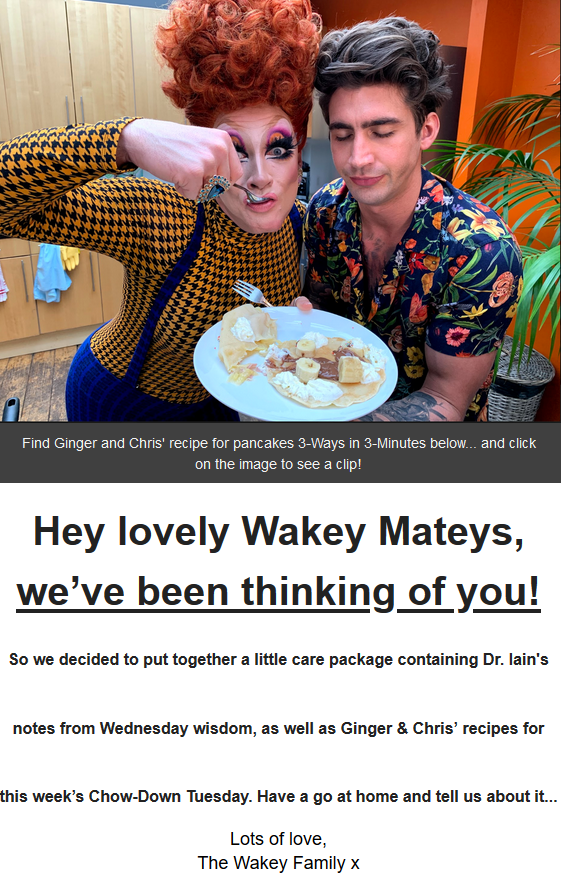


- a progress page for users that provided feedback. We decided to not present the changes in health outcomes in the progress page as people with negative thoughts can become preoccupied with this.


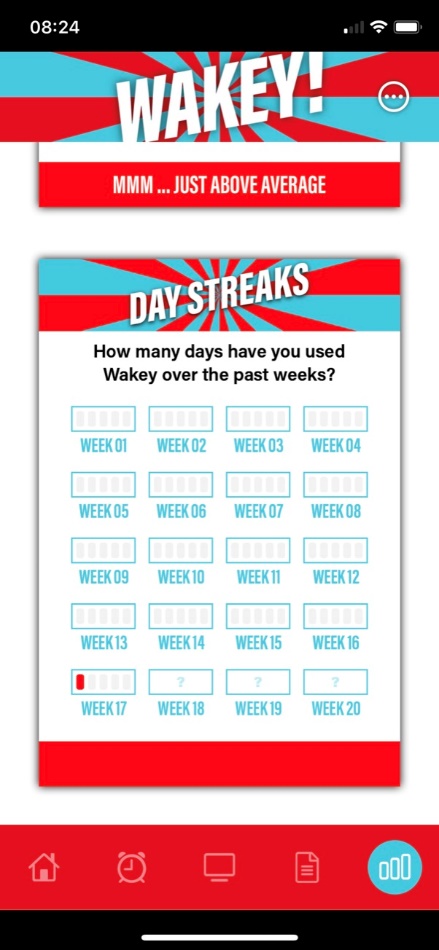

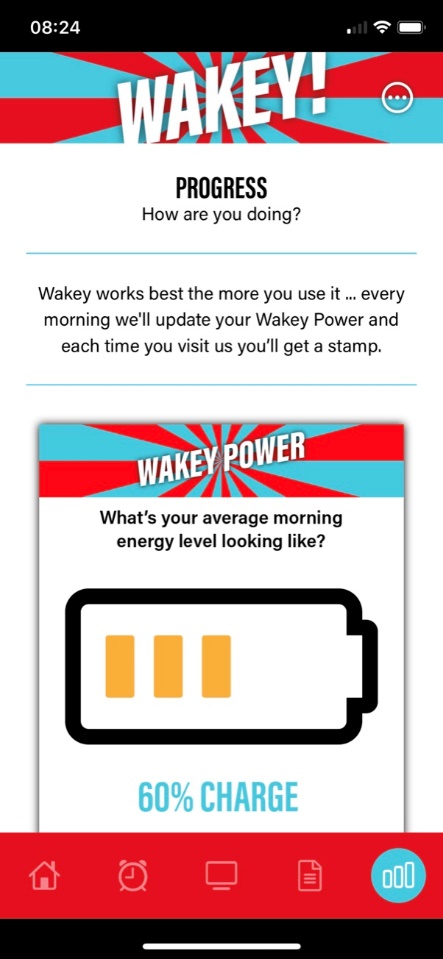


# Terms and Conditions


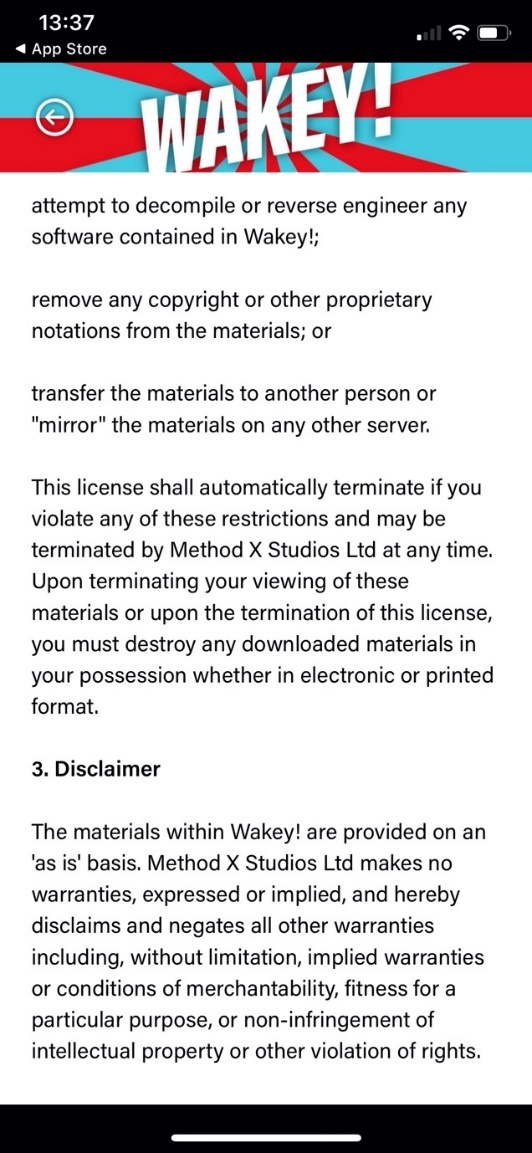

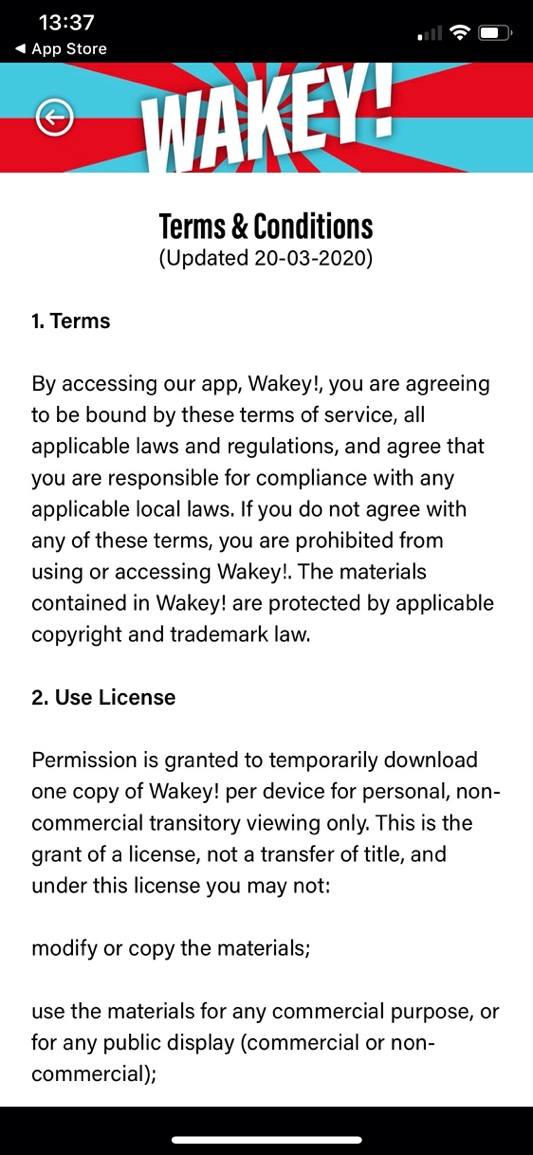


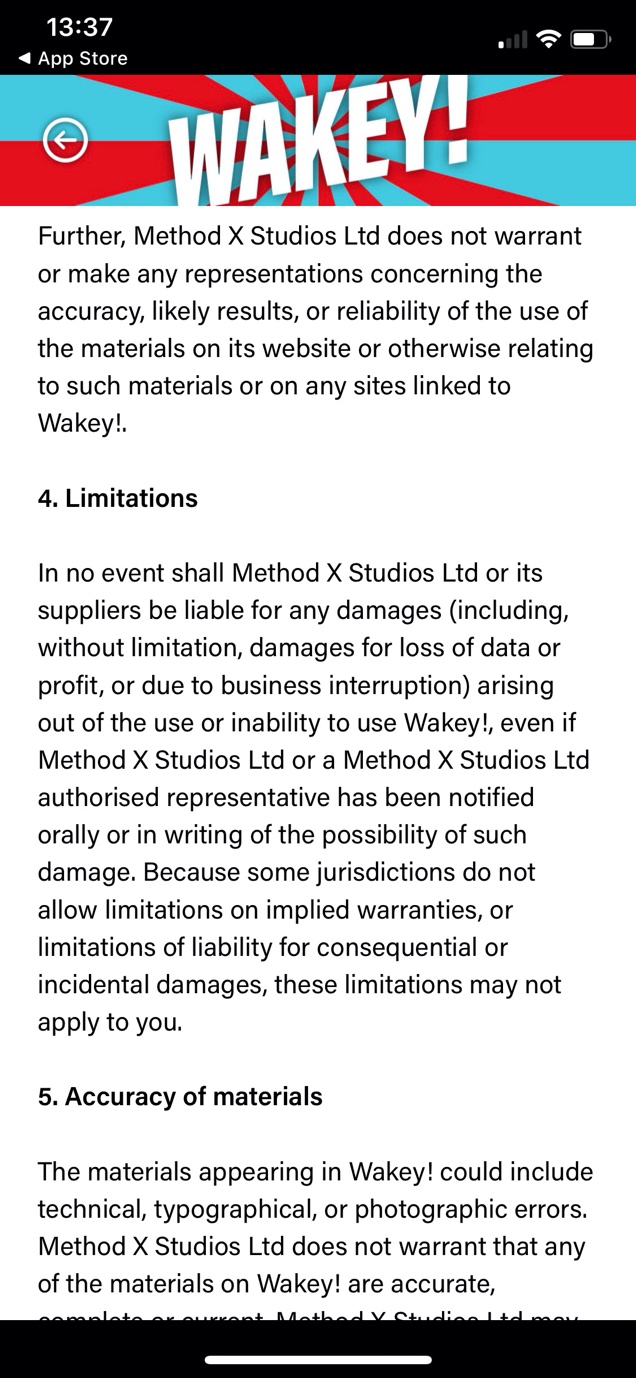

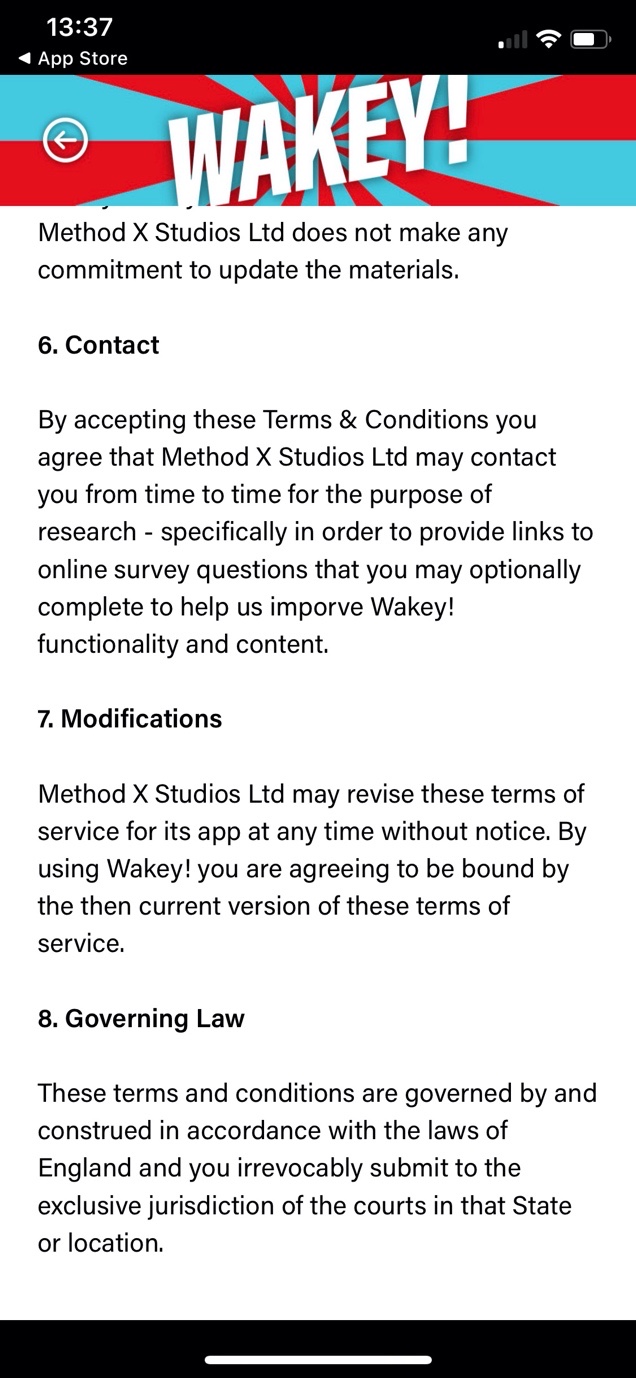


# Privacy Policy


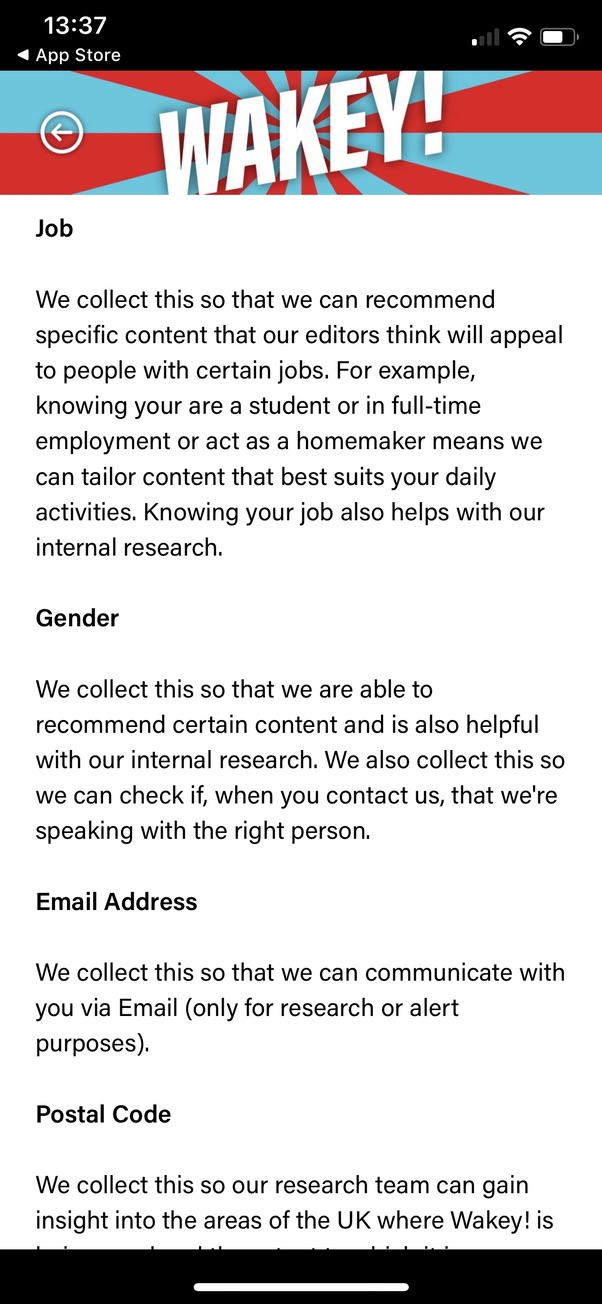

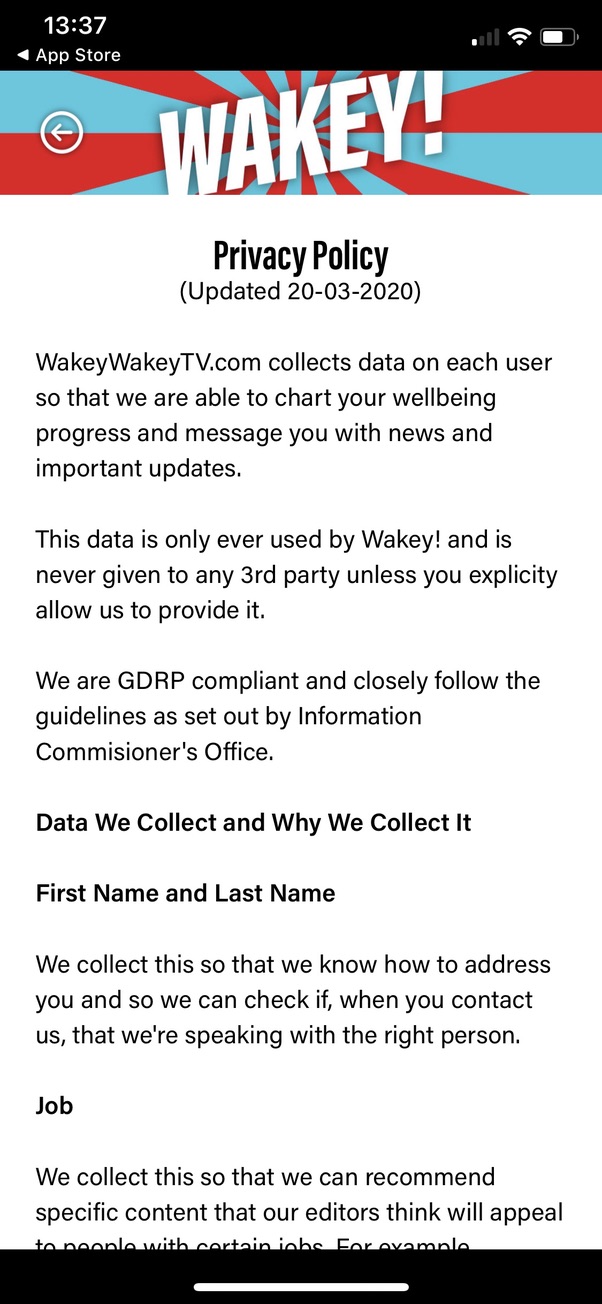


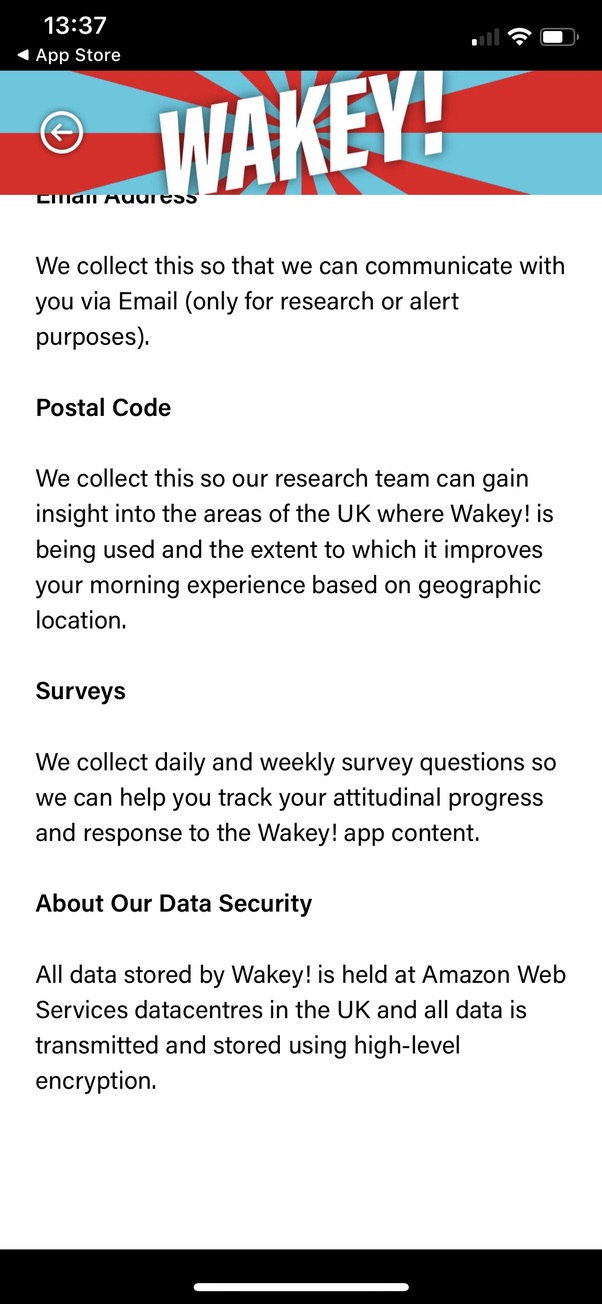


# Personal data collection

The following data was collected from users when they were registering:

gender

male, female, non-binary, other, prefer not to say

age range

under 18, 18–24, 25–34, 35–44, 45–54, 55–64, 65 and older, prefer not to say

occupation

caregiver, looking after family/home, retired, sickness/disability, student, unemployed, prefer not to say or occupation from the following list:

Account Executive, Account Manager, Accountant, Accounts Assistant, Administrator, Analyst, Architect, Armed Forces, Auditor, Barrister, Business Analyst, Business Development Manager, Buyer, Commercial Manager, Cleaner, Consultant, Credit Controller, Dentist, Design Engineer, Designer, Developer, Doctor, Driver, Editor, Electrician, Engineer, Estimator, Finance Director, Finance Manager, Financial Analyst, Financial Controller, GP, Graphic Designer, HR Manager, IT Manager, Lawyer, Lecturer, Management Accountant, Managing Director, Marketing Director, Marketing Manager, Mechanical Engineer, Nurse, Office Manager, Operations Manager, Personal Assistant, Pharmacist, Pilot, Plumber, Product Manager, Production Manager, Programmer, Project Engineer, Project Manager, Quantity Surveyor, Receptionist, Recruitment Consultant, Researcher, Sales Assistant, Sales Director, Sales Manager, Scientist, Secretary, Social Worker, Software Developer, Solicitor, Store Manager, Teacher, Technician, Trader, Trainer, Web Designer, Web Developer

The four ONS well-being questions that are used in the Annual Population Survey (ONS, 2018) were rated on a scale of 0 (“not at all”) to 10 (“completely”)

- “Overall, how satisfied are you with your life nowadays?“
- “Overall, to what extent do you feel that the things you do in your life are worthwhile?“
- “Overall, how happy did you feel yesterday?“
- “Overall, how anxious did you feel yesterday?“

Self-efficacy was measured by asking users to assess to what extent they agreed with the statement “I can successfully overcome life’s daily challenges”. The answers included: 1 strongly disagree, 2 disagree, 3 neither agree or disagree, 4 agree, 5 strongly agree.

The sixth question asked users to rate how easy it was to get up in the current morning on a scale of 0 (“not at all”) to 10 (“completely”) (users were also asked this question on a daily basis).

# Socio-economic groups based on occupation

Users were divided into different socio-economic groups based on the National Statistics Socio-economic classification (Table S1).

Table S1. List of occupations by NS-SEC analytic class

| **NS-SEC analytic class** | **Occupations** |
| --- | --- |
| 1. Higher managerial, administrative and professional occupations (high socio-economic status) | Account Manager  Accountant  Architect  Barrister  Business Analyst  Business Development Manager  Commercial Manager  Dentist  Design Engineer  Developer  Doctor  Finance Director  Finance Manager  Financial Controller  GP  HR Manager  IT Manager  Lawyer  Lecturer  Management Accountant  Managing Director  Marketing Director  Marketing Manager  Operations Manager  Pharmacist  Pilot  Product Manager  Production Manager  Programmer  Project Engineer  Project Manager  Researcher  Sales Director  Scientist  Software Developer  Solicitor |
| 1. Lower managerial, administrative and professional occupations (high socio-economic status) | Account Executive  Analyst  Buyer  Consultant  Editor  Estimator  Financial Analyst  Nurse  Office Manager  Quantity Surveyor  Recruitment Consultant  Sales Manager  Social Worker  Store Manager  Teacher  Technician  Trainer  Web Designer  Web Developer |
| 1. Intermediate occupations (middle socio-economic status) | Accounts Assistant  Administrator  Armed Forces  Auditor  Credit Controller  Graphic Designer  Personal Assistant  Secretary |
| 1. Small employers and own account workers (middle socio-economic status) | Designer  Trader |
| 1. Lower supervisory and technical occupations (middle socio-economic status) | Electrician  Engineer  Mechanical Engineer  Plumber |
| 1. Semi-routine occupations (low socio-economic status) | Receptionist  Sales Assistant |
| 1. Routine occupations (low socio-economic status) | Cleaner  Driver |
| 1. Not classified elsewhere | Caregiver  Looking after family, home/Homemaker  Sickness/disability  Retired  Student  Graduate  Unemployed |

# The App – Home page


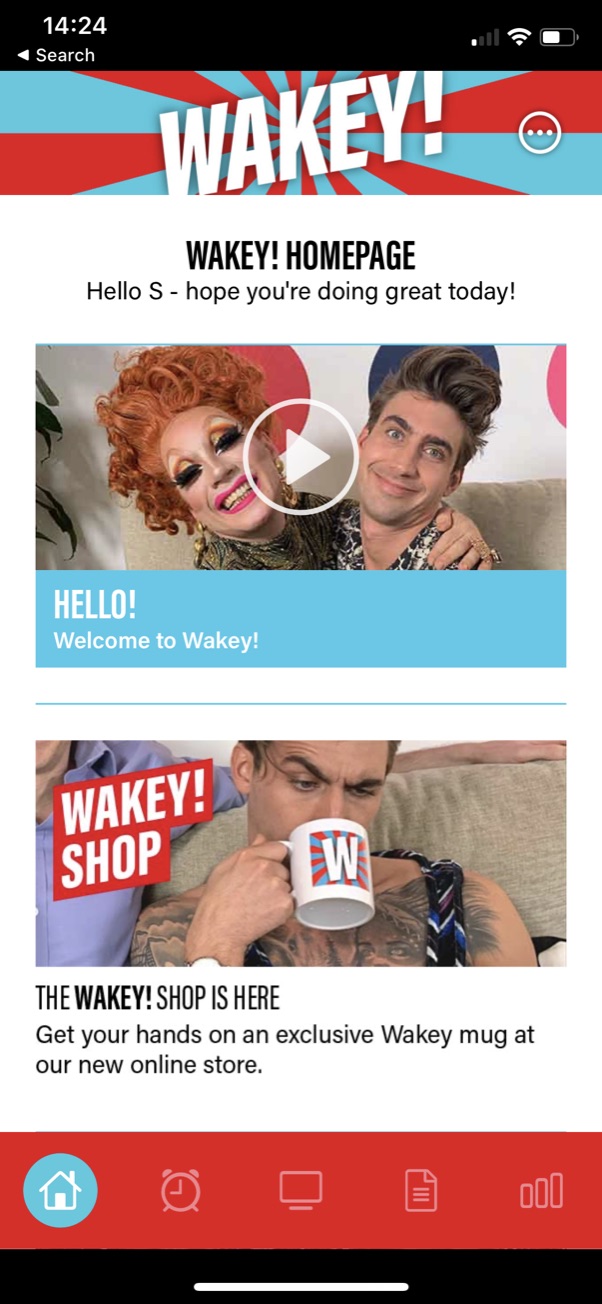


# Qualitative study procedures

Users were divided into three different groups depending on their engagement:

- inactive (had seen maximum of two episodes or the Welcome video),
- became inactive (had seen minimum of three episodes on their onboarding week and thereafter maximum two episodes per week),
- very engaged (had seen at least 60% of the episodes).

While the ideal number of participants in a qualitative study varies across disciplines and researchers, we took a pragmatic approach to the sample size – aiming to have an equal number of interviewees in each group – and did not continue to interview to data saturation due to limitations in the number of responses and resources available. Initially, 21 randomly selected participants across different genders and age range (7 from each of the three groups) were sent emails and invited to take part in the study.

As the response rate was expected to be low, invitations were sent to an additional eight users with similar characteristics, and all invitees received push notifications in the app. Due to a very low response rate, invitations (via Mailchimp) and push notifications (via app) were sent to everyone who fit the criteria of belonging to target socio-economic group (n=1524). This time an additional group (irregular users) was included. Everyone received a maximum of two push notifications and two emails (second one as a reminder). For the sampling in the qualitative study, the occupations were placed in higher (ABC1) or lower (C2DE), or social grade not known categories outlined by McDonald & Dunbar (McDonald & Dunbar, 2004).

Table S2: Occupations’ distribution by social grade

| **ABC1** | | **C2DE** |
| --- | --- | --- |
| Account Executive  Account Manager  Accountant  Accounts Assistant  Administrator  Analyst  Architect  Armed Forces  Auditor  Barrister  Business Analyst  Business Development Manager  Buyer  Commercial Manager  Consultant  Credit Controller  Dentist  Design Engineer  Designer  Developer  Doctor  Editor  Engineer  Estimator  Finance Director  Finance Manager  Financial Analyst  Financial Controller  GP  Graduate  Graphic Designer  HR Manager  IT Manager  Lawyer  Lecturer  Management Accountant  Managing Director  Marketing Director | Marketing Manager  Mechanical Engineer  Nurse  Office Manager  Operations Manager  Personal Assistant  Pharmacist  Pilot  Product Manager  Production Manager  Programmer  Project Engineer  Project Manager  Quantity Surveyor  Receptionist  Recruitment Consultant  Researcher  Sales Director  Sales Manager  Scientist  Secretary  Social Worker  Software Developer  Solicitor  Store Manager  Teacher  Technician  Trader  Trainer  Web Designer  Web Developer | Cleaner  Driver  Electrician  Plumber  Sales Assistant |
|  |  | **Social grade not known** |
|  |  | Caregiver  Homemaker  Looking after family/ home  Retired  Unemployed  Sickness/ disability  Student |

#

# Interview topic guide

*Background*

Could you please tell me a bit about yourself? Prompt - whether they are in paid employment, religious and cultural practices.

As Wakey! aims to improve people’s mood, how would you rate your mental well-being (any issues with stress/mental health). What kind of things have you done or do now to improve your mood?

*Experience of using Wakey! (regular users)*

How did you hear about Wakey!? What about it (advert, people’s descriptions, etc) made you interested in the app?

How often did you use Wakey!? Did you watch live stream and/or archived episodes? What do you think of the option of having both?

Has your watching changed during the past few weeks (lockdown)?

How did you find Wakey! fitted into your morning routine?

What has been your experience of using Wakey! so far?

- technical issues (e.g. chat, alarm, loading videos)
- social elements (e.g. the ability to communicate on social platforms – FB, Instagram, Twitter)
- presenters (e.g. Ginger, Chris, Dr. Iain, special guests)
- content (e.g. topics covered, different sections – old news, bed aerobics, prescribed sleep content)
- pace of the show
- the set
- length
- features (e.g. chat, Q&A, challenges and competitions, newsletters, articles, progress page, push notifications, quiz)

What did you think of the questions you were asked daily and weekly? Did you answer regularly? How so? Probe - personal relevance, frequency, burden too high.

Any questions you would prefer to see/ would like to be asked or track? Would you like to have the possibility to enter your own questions that you can track weekly?

Can you tell me about any changes there might have been to your daily life / morning routines since you started using Wakey!? (e.g. any changes you have made?)

If yes: Can you tell me a bit more about those changes? Can you tell me about what made you make those changes? Why now?

Have you noticed any positive/negative changes in mood (e.g. more positive), sleep (e.g. better), anything else that might relate to using the app?

*For those who discontinued using Wakey!*

How did you hear about Wakey!? What about it (advert, people’s descriptions, etc) made you interested in the app?

Could you describe your experience of using Wakey!? Anything you really liked and/or disliked?

Could you tell us a bit about why you stopped using Wakey! after a short while of using it?

What did you think of the questions you were asked daily and weekly? Did you answer regularly? How so?

Any questions you would prefer to see/ would like to be asked or track? Would you like to have the possibility to enter your own questions that you can track weekly?

*Future improvements*

Imagine that Wakey! was going to be changed and you can decide on up to three things that stay unchanged. What would you keep?

Now imagine the opposite and you can exclude up to three things while everything else stays the same. What would you exclude?

Can you think of any other ways that we can make Wakey! better in the future?

Prompt - connection with wearable data like sleep or heart rate monitor, any other apps they use that really help with sleep/well-being that they like features of. Add-ons.

*Other issues*

Any other issues you would like to raise.
