# Supplementary material for: Improving Access to Behavioral Strategies to Improve Mental Well-being With an Entertaining Breakfast Show App: Feasibility Evaluation Study
Source: JMIR Form Res. 2022 Mar 23;6(3):e25715. doi: 10.2196/25715 (PMC8987957; doi:10.2196/25715)
Supplement: Multimedia Appendix 2 [file formative_v6i3e25715_app2.docx]

**Multimedia Appendix 2 – Supporting tables**

Table S1. All variables included in analysis predicting characteristics of active users and improvements in well-being outcomes.

| Predictor | Variable type | Predictors of active user | Predictors of improvements in well-being outcomes | | | | | |
| --- | --- | --- | --- | --- | --- | --- | --- | --- |
|  |  |  | ONS 1 | ONS 2 | ONS 3 | ONS 4 | Sleep quality | Self-efficacy |
| **Socio-demographic characteristics** | | | | | | | | |
| Age range | Category:   - 18–24 - 25–34 - 35–44 - 45–54 - 55–64 - 65 or older | ✓ | ✓ | ✓ | ✓ | ✓ | ✓ | ✓ |
| Gender | Category:   - Male - Female - Non-binary/ other | ✓ | ✓ | ✓ | ✓ | ✓ | ✓ | ✓ |
| Occupation type | - Caregiver - Looking after family, home - Retired - Sickness, disability - Student - Unemployed - Employed | ✓ | ✓ | ✓ | ✓ | ✓ | ✓ | ✓ |
| Socio-economic status | - High - Middle - Low - Not classified elsewhere | ✓ | ✓ | ✓ | ✓ | ✓ | ✓ | ✓ |
| **Engagement with the App** | | | | | | | | |
| How many weeks ago the user joined | Continuous 1–12 | ✓ | ✓ | ✓ | ✓ | ✓ | ✓ | ✓ |
| On which day of the week the user joined | Categorical:  Monday-Friday | ✓ | ✓ | ✓ | ✓ | ✓ | ✓ | ✓ |
| Number of weekdays user had access to Wakey! | Continuous | ✖ | ✓ | ✓ | ✓ | ✓ | ✓ | ✓ |
| No of days between baseline and the last daily answer | Continuous | ✖ | ✓ | ✓ | ✓ | ✓ | ✓ | ✓ |
| No of days between baseline and the last weekly answer | Continuous | ✖ | ✓ | ✓ | ✓ | ✓ | ✓ | ✓ |
| On how many days did the user answer daily questions | Continuous | ✖ | ✓ | ✓ | ✓ | ✓ | ✓ | ✓ |
| **Engagement with the App’s content** | | | | | | | | |
| How many live or arch episodes the user saw in total | Continuous | ✖ | ✓ | ✓ | ✓ | ✓ | ✓ | ✓ |
| How many live episodes the user saw in total | Continuous | ✖ | ✓ | ✓ | ✓ | ✓ | ✓ | ✓ |
| How many archived episodes the user saw in total | Continuous | ✖ | ✓ | ✓ | ✓ | ✓ | ✓ | ✓ |
| Segmentation of type of user | Category:   - inactive - became inactive - irregular - engaged | ✖ | ✓ | ✓ | ✓ | ✓ | ✓ | ✓ |
| **Entertainment value of episodes and ease of getting up in the morning** | | | | | | | | |
| User’s average of all of his/her responses on ease of getting up in the morning | Mean of all responses | ✓ | ✓ | ✓ | ✓ | ✓ | ✓ | ✓ |
| User’s average of all of his/her responses on the entertainment value of episode | Mean of all responses | ✓ | ✓ | ✓ | ✓ | ✓ | ✓ | ✓ |
| **Baseline ONS well-being questions scale** |  |  |  |  |  |  |  |  |
| ONS 1: Overall, how satisfied are you with your life nowadays? | Likert scale: 0-10  0 (“not at all”) to 10 (“completely”) | ✓ | ✖ | ✖ | ✖ | ✖ | ✓ | ✓ |
| ONS 2: Overall, to what extent do you feel that the things you do in your life are worthwhile | Likert scale: 0-10  0 (“not at all”) to 10 (“completely”) | ✓ | ✖ | ✖ | ✖ | ✖ | ✓ | ✓ |
| ONS 3: Overall, how happy did you feel yesterday? | Likert scale: 0-10  0 (“not at all”) to 10 (“completely”) | ✓ | ✖ | ✖ | ✖ | ✖ | ✓ | ✓ |
| ONS 4: Overall, how anxious did you feel yesterday? | Likert scale: 0-10  0 (“very low”) to 10 (high) | ✓ | ✖ | ✖ | ✖ | ✖ | ✓ | ✓ |
| **Baseline sleep and self-efficacy questions** | | | | | | | | |
| Sleep quality: How easy was it to get up this morning? | Likert scale: 0-10  (“not at all”) to 10 (“completely”) | ✓ | ✓ | ✓ | ✓ | ✓ | ✖ | ✓ |
| Self-efficacy: I can successfully overcome life’s daily challenges | Likert scale: 1-5 –  strongly disagree, disagree, neither agree or disagree, agree, strongly agree | ✓ | ✓ | ✓ | ✓ | ✓ | ✓ | ✖ |

Table S2. All variables included in analysis predicting characteristics of engaged users (watched ≥20% of available episodes).

| Predictor | Variable predicted being an engaged user | OR^a^ and 95% CI^b^ provided where P<.05) |
| --- | --- | --- |
|  |  |  |
| **Socio-demographic characteristics** | | |
| Age range | X | *P*=.12 |
| Gender | X | *P*=.60 |
| Occupation type | X | *P*=.26 |
| Socio-economic group | X | *P*=.10 |
| **Variables tracking participant movement through the study** | | |
| How many weeks ago the user joined | ✓ | OR 0.92 (CI 0.88-0.96) *P*<.001 |
| On which day of the week the user joined | X | *P*=.51 |
| **Entertainment value and ease of getting up in the morning^c^, scale of 0-10 scale** | | |
| User’s average of all his/her responses on ease of getting up in the morning | ✓ | OR 1.15 (CI 1.11-1.20) *P*<.001 |
| User’s average of all his/her responses on the entertainment value of episode | ✓ | OR 1.16 (CI 1.12-1.20) *P*<.001 |
| **Baseline ONS well-being questions scale of 0-10** | | |
| ONS 1: Overall, how satisfied are you with your life nowadays? | X | *P*=.12 |
| ONS2: Overall, to what extent do you feel that the things you do in your life are worthwhile | X | *P*=.13 |
| ONS 3: Overall, how happy did you feel yesterday? | X | *P*=.61 |
| ONS 4: Overall, how anxious did you feel yesterday? | X | *P*=.81 |
| **Baseline sleep and self-efficacy questions** | | |
| Sleep quality: How easy was it to get up this morning? | ✓ | OR 1.06 (CI 1.03-1.10) *P*=.001 |
| Self-efficacy: I can successfully overcome life’s daily challenges | X | *P*=.54 |

*^a^OR – odds ratio, ^b^CI – confidence interval, ^c^asked daily after the episode*

Table S3. Socio-demographic and socio-economic characteristics among engaged (watched ≥20% of available episodes) and not engaged (watched <20% of available episodes) users.

|  | Engaged | Not engaged |  |
| --- | --- | --- | --- |
|  | n/N (%) | n/N (%) |  |
| **Gender^1^** | | | |
| Female | 266/429 (62.0) | 2223/3464 (64.2) | *P*=.38 |
| Male | 163/429 (38.0) | 1241/3464 (35.8) |  |
| **Age^2^** | | | |
| 18–24 | 51/435 (11.7) | 592/3537 (16.7) | *P*=.15 |
| 25–34 | 146/435 (33.6) | 1122/3537 (31.7) |  |
| 35–44 | 133/435 (30.6) | 1062/3537 (30.0) |  |
| 45–54 | 81/435 (18.6) | 608/3537 (17.2) |  |
| 55 or older | 24/435 (5.5) | 153/3537 (4.3) |  |
| **Socio-economic group** | | | |
| High | 164/371 (44.2) | 1298/3084 (42.1) | *P*=.10 |
| Middle | 56/371 (15.1) | 358/3084 (11.6) |  |
| Low | 30/371 (8.1) | 260/3084 (8.4) |  |
| Not classified elsewhere | 121/371 (32.6) | 1168/3084 (37.9) |  |

*^1^As the number of non-binary users who were engaged users was very small, they were excluded from this comparison. ^2^As the number of engaged users who were older than 65 was very small, this group was combined with the age group of 55–64-year-olds.*

Table S4. Multivariate logistic regression model of predictors of being an engaged user (watched ≥20% of available episodes).

| Predictor | OR^a^ (95% CI^b^) |  |
| --- | --- | --- |
| Average score for how entertaining the daily episodes were | 1.16 (1.12-1.20) | *P*<.001 |
| Sleep score at baseline (range 0-10) | 1.07 (1.02-1.12) | *P*=.002 |
| How many weeks ago the user joined (3-12) | 0.90 (0.85-0.95) | *P*<.001 |

*^a^OR – odds ratio, ^b^CI – confidence interval*

Table S5. Socio-demographic and socio-economic characteristics among active users who did and did not provide follow-up data (ONS and sleep indicators).

|  | Provided follow-up data | Did not provide follow-up data |  |
| --- | --- | --- | --- |
|  | n/N (%) | n/N (%) |  |
| **Gender^1^** | | | |
| Female | 139/227 (61.2) | 2300/3565 (64.5) | *P*=.32 |
| Male | 88/227 (38.8) | 1265/3565 (35.5) |  |
| **Age^2^** | | | |
| 18–24 | 30/229 (13.1) | 599/3639 (16.5) | *P*=.37 |
| 25–34 | 71/229 (31.0) | 1159/3639 (31.9) |  |
| 35–44 | 67/229 (29.3) | 1100/3639 (30.2) |  |
| 45–54 | 48/229 (21.0) | 623/3639 (17.1) |  |
| 55 or older | 13/229 (5.7) | 158/3639 (4.3) |  |
| **Socio-economic group** | | | |
| High | 75/201 (37.3) | 1350/3170 (42.6) | *P*=.25^3^ |
| Middle | 32/201 (15.9) | 371/3170 (11.7) |  |
| Low | 17/201 (8.5) | 268/3170 (8.5) |  |
| Not classified elsewhere | 77/201 (38.3) | 1181/3170 (37.3) |  |

*^1^As the number of non-binary users who provided follow-up data was very small, they were excluded from this comparison. ^2^As the number of users who were older than 65 and who provided follow-up data was very small, this group was combined with the age group of 55–64-year-olds. ^3^There is a statistically significant difference between the first two groups (P=.045).*

Table S6. Socio-demographic and socio-economic characteristics among users who did and did not provide follow-up data (self-efficacy indicator).

|  | Provided follow-up data | Did not provide follow-up data |  |
| --- | --- | --- | --- |
|  | n/N (%) | n/N (%) |  |
| **Gender^1^** | | | |
| Female | 96/155 (61.9) | 2343/3637 (64.4) | *P*=.53 |
| Male | 59/155 (38.1) | 1294/3637 (35.6) |  |
| **Age^2^** | | | |
| 18–24 | 19/157 (12.1) | 610/3711 (16.4) | *P*=.03 |
| 25–34 | 47/157 (29.9) | 1183/3711 (31.9) |  |
| 35–44 | 42/157 (26.8) | 1125/3711(30.3) |  |
| 45–54 | 36/157 (22.9) | 635/3711 (17.1) |  |
| 55 or older | 13/157 (8.3) | 158/3711 (4.3) |  |
| **Socio-economic group** | | | |
| High | 47/140 (33.6) | 1378/3231 (42.7) | *P*=.12^3^ |
| Middle | 23/140 (16.4) | 380/3231 (11.8) |  |
| Low | 14/140 (10.0) | 271/3231 (8.4) |  |
| Not classified elsewhere | 56/140 (40.0) | 1202/3231 (37.2) |  |

*^1^As the number of non-binary users who provided follow-up data was very small, they were excluded from this comparison. ^2^As the number of users who were older than 65 and who provided follow-up data was very small, this group was combined with the age group of 55–64-year-olds. ^3^There is a statistically significant difference between the first two groups (P=.03).*

Table S7. Univariable associations of variables with improvements in well-being outcomes (>=1 point).

| Predictor | Predictors of improvements in health outcomes (OR and 95% CI provided where *P*<.05) | | | |
| --- | --- | --- | --- | --- |
|  | ONS 1: Life satisfaction | ONS 2: Worthwhile | Sleep quality | Self-efficacy |
| Age Range | *P*=.64 | *P*=.98 | *P*=.13 | *P*=.62 |
| Gender | *P*=.86 | *P*=.30 | *P*=.34 | *P*=.96 |
| Socio-economic status | *P*=.23 | *P*=.17 | *P*=.96 | *P*=.88 |
| How many weeks ago the participant entered the study | *P*=.29 | *P*=.74 | *P*=.91 | *P*=.30 |
| On which day of the week the user joined | *P*=.07 | *P*=.64 | *P*=.86 | *P*=.68 |
| Number of weekdays user had access to Wakey! | *P*=.20 | *P*=.84 | *P*=.91 | *P*=.24 |
| No of days between baseline and the last daily answer | *P*=.25 | *P*=.42 | *P*=.52 | *P*=.43 |
| Last week they provided follow-up data | *P*=.40 | *P*=.19 | *P*=.32 | *P*=.80 |
| No of days between baseline and the last weekly answer | *P*=.40 | *P*=.16 | *P*=.30 | *P*=.76 |
| On how many days did the user answer daily questions | *P*=.36 | *P*=.80 | *P*=.18 | *P*=.77 |
| How many live or arch episodes the user saw in total | *P*=.46 | *P*=.68 | *P*=.08 | *P*=.63 |
| How many live episodes the user saw in total | *P*=.38 | *P*=.98 | *P*=.28 | *P*=.57 |
| How many archived episodes the user saw in total | *P*=.98 | *P*=.32 | OR 1.04 (CI 1.00-1.08) *P*=.04 | *P*=.95 |
| Segmentation of type of user | *P*=.49 | *P*=.61 | *P*=.19 | *P*=.91 |
| Engaged user (>=20% available episodes) | *P*=.96 | *P*=.60 | *P*=.45 | *P*=.44 |
| User’s average of all his/her responses on ease of getting up in the morning | *P*=.11 | *P*=.53 | *P*=.52 | *P*=.73 |
| User’s average of all his/her responses on the entertainment value of episode | *P*=.22 | *P*=.90 | *P*=.23 | *P*=.92 |
| ONS 1: Overall, how satisfied are you with your life nowadays? | NA | NA | OR 0.83 (CI 0.74-0.93) *P*=.002 | *P*=.12 |
| ONS 2: Overall, to what extent do you feel that the things you do in your life are worthwhile | NA | NA | OR 0.77 (CI 0.69-0.87) *P*<.001 | OR 0.86 (CI 0.76-0.99) *P*=.03 |
| ONS 3: Overall, how happy did you feel yesterday? | NA | NA | OR 0.85 (CI 0.76-0.94) *P*= .003 | *P*=.69 |
| ONS 4: Overall, how anxious did you feel yesterday? | NA | NA | *P*=.99 | *P*=.73 |
| Sleep quality: How easy was it to get up this morning? | *P*=.82 | OR 0.89 (CI 0.81-0.97) *P*=.01 | NA | *P*=.58 |
| Self-efficacy: I can successfully overcome life’s daily challenges | OR 0.63 (CI 0.50-0.80) *P*<.001 | OR 0.78 (CI 0.63-0.98) *P*=.03 | 0.24 | NA |

Table S8. Multivariate logistic models predicting improvements (>=1 point improvement) in well-being outcomes.

|  | Number of users included in model | Results |
| --- | --- | --- |
| Sleep: How easy was it to get up this morning? (0-10) | 230 | Total archived episodes watched:  OR 1.05 (CI 1.01-1.10) *P*=.012  ONS2 Life is worthwhile:  OR 0.75 (CI 0.67-0.86) *P*<.001 |
